# Structured Onboarding of International Nurses in an Italian Healthcare Organization: An Exploratory Multi-Stakeholder Pilot and Feasibility Observational Study

**DOI:** 10.3390/healthcare14162557

**Published:** 2026-08-15

**Authors:** Marcello Torre, Rosario Caruso, Antonio Maria Giuseppe Staffa, Cristina Arrigoni, Arianna Magon

**Affiliations:** 1 Department of Biomedicine and Prevention, University of Rome Tor Vergata, 00133 Rome, Italy; marcello.torre@asst-settelaghi.it; 2 Direction of Healthcare Professions, Azienda Socio Sanitaria Territoriale Sette Laghi, 21100 Varese, Italy; antoniomariagiuseppe.staffa@asst-settelaghi.it; 3 Department of Biomedical Sciences for Health, University of Milan, 20133 Milan, Italy; 4 Health Professions Research and Evidence Transfer Unit, IRCCS MultiMedica, 20099 Sesto San Giovanni, Italy; 5 Department of Public Health, Experimental and Forensic Medicine, Section of Hygiene, University of Pavia, 27100 Pavia, Italy; cristina.arrigoni@unipv.it (C.A.); arianna.magon@unipv.it (A.M.)

**Keywords:** international nurses, nurse migration, onboarding, pilot study, feasibility study, retention, welcome, observational study

## Abstract

**Highlights:**

**What are the main findings?**

- The study, among the first in Italy on this topic, demonstrates the feasibility of a multi-stakeholder evaluation of a structured onboarding pathway in a non-English-speaking Italian healthcare setting.
- Response-level ratings showed more favourable patterns at later conceptual timepoints in perceived language proficiency and professional training/competence, although these were anonymous, partly proxy-rated measures with only T0–T1 pairable within response rows.

**What are the implications of the main findings?**

- Future studies should collect stable pseudonymized identifiers, objective language and professional indicators, validated measures, and aggregate baseline descriptors permitted by privacy rules.
- The main feasibility finding is the data infrastructure required for a confirmatory longitudinal evaluation.

**Abstract:**

**Background/Objectives**: International nurse recruitment is increasingly important, but early integration depends on language, professional adaptation, organizational welcome, and retention support. Evidence from non-English-speaking healthcare systems and multi-stakeholder implementation evaluations remains limited. This study aimed to evaluate a structured onboarding pathway as an exploratory, response-level pilot and a feasibility platform. **Methods**: Anonymous five-point Likert questionnaires were administered to clinical coaches, ward teams, and newly hired international nurses at conceptual time points T0, T1, and T2. T0 was partly retrospective because the first survey wave, administered at approximately 3 months, asked respondents to rate both arrival status and current status. Analyses were response-level; T0–T1 ratings were summarized with a paired within-row sensitivity analysis when both ratings were available, whereas T2 could not be linked to the earlier wave. **Results:** The primary dataset contained 410 anonymous response-level observations, mostly from ward-team proxy ratings. Median language proficiency ratings were 3 (IQR 2–3) at T0, 3 (IQR 3–4) at T1, and 3 (IQR 3–4) at T2; the proportion of ratings at or above the pragmatic adequacy threshold (≥3) rose from 53.5% to 76.4% and 91.0%, respectively. For professional training/competence, median ratings were 3 (IQR 2–4), 3 (IQR 3–4), and 3 (IQR 3–4), with corresponding adequacy-threshold proportions of 53.5%, 78.0%, and 89.7%. However, stakeholder-specific patterns differed, and international nurses’ self-rated language proficiency followed a non-monotonic pattern. In the nurse-specific analytic subset (14 anonymous response rows), overall welcome was associated with intention to stay (Spearman rho = 0.73, FDR-adjusted *p* = 0.016) and willingness to recommend the experience (rho = 0.86, FDR-adjusted *p* = 0.002). **Conclusions:** The pilot generated hypotheses and, more importantly, mapped feasibility requirements for a future confirmatory study. Although the findings should not be interpreted causally or as paired longitudinal evidence, they provide an initial overview of issues related to the integration of international nurses in non-English-speaking healthcare settings. A future study should prospectively collect pseudonymized identifiers, objective and validated measures, and a pre-specified longitudinal analytic model.

## 1. Introduction

Health systems in Italy are facing persistent nursing workforce shortages and are increasingly considering international recruitment as a strategy to sustain service capacity [1]. In this context, internationally educated nurses may contribute to workforce stabilization, but their integration into the Italian National Health Service requires more than recruitment alone [2]. Entry into a non-English-speaking healthcare system involves specific challenges, including Italian-language communication, adaptation to local professional norms, recognition of prior qualifications, and integration within ward teams [3]. Evidence from the Italian context remains limited, particularly regarding structured organizational pathways designed to support the early onboarding of international nurses.

This Italian experience should be interpreted within a broader global workforce context. International mobility of nurses has increased as high-income health systems respond to persistent workforce gaps, while recent global and Organisation for Economic Co-operation and Development (OECD) reporting highlights the continuing mismatch between nursing workforce supply and demand [4,5]. International recruitment could therefore be a pragmatic response to service pressure, but it does not, by itself, ensure professional integration, retention, or safe transition into local clinical practice [6].

The post-arrival period is shaped by language barriers, cultural adjustment, professional role adaptation, recognition of prior competence, and organizational support [7,8,9,10,11,12,13]. These barriers are especially salient during the first months after entry, when transition shock and intention to leave may emerge among newly hired nurses [14]. Early onboarding is, therefore, not only an administrative process but also a retention-sensitive intervention window [7].

Three gaps motivate this study. First, much of the evidence on internationally educated nurses comes from English-speaking destination systems, whereas integration in non-English-speaking settings entails different language and professional recognition demands [6]. Second, most studies privilege the migrant nurse perspective, while fewer capture coach, ward-team, and nurse perspectives simultaneously [15]. This distinction is important because integration is both an individual experience and an organizational process involving local supervisors, colleagues, and receiving institutions. Third, many publications describe barriers, but fewer evaluate a real structured onboarding pathway while also testing whether routine organizational data can support future confirmatory research [6,16]. Recent work on transition programmes for newly hired nurses also highlights conceptual plurality in this field, strengthening the need for transparent descriptions of what is being implemented and measured [7].

These gaps indicate a lack of empirical evidence on structured onboarding pathways for international nurses in non-English-speaking healthcare systems, particularly studies that combine a multi-stakeholder perspective with an explicit assessment of data feasibility for future longitudinal research. This gap is important because successful integration is not only an individual process experienced by internationally educated nurses, but also an organizational and relational process involving local teams, supervisors, and the broader receiving institution. In addition, without feasible data structures that allow repeated observations to be linked over time, healthcare organizations cannot determine whether onboarding pathways can be evaluated rigorously in relation to perceived integration, welcome, and retention-related outcomes [17].

In this regard, the present study uses a structured, language-aware onboarding pathway implemented in an Italian healthcare organization as a real-world feasibility platform. Rather than estimating causal effectiveness, the study examines whether routinely collected multi-stakeholder data can describe perceived integration trajectories and inform the design of a future confirmatory longitudinal evaluation [11,12,18,19]. In this framework, coach and ward-team ratings are interpreted as organizational perceptions of integration, not as objective measures of nurses’ competence. Perceived welcome was included as a theoretically relevant and potentially modifiable dimension of onboarding, reflecting the extent to which international nurses felt recognized, supported, and included within the receiving organization. Therefore, this study aimed to: (1) describe exploratory response-level patterns in perceived language proficiency and professional training/competence across the onboarding period; (2) explore welcome and retention-related outcomes among international nurses; (3) assess whether the available data structure was adequate for longitudinal evaluation, including denominators, missingness, and linkage constraints; and (4) identify planning assumptions and methodological requirements for a future confirmatory study.

## 2. Materials and Methods

### 2.1. Design and Reporting Framework

This was an exploratory, anonymous response-level observational pilot and feasibility evaluation of a structured onboarding pathway for international nurses. Reporting was aligned with the Strengthening the Reporting of Observational Studies in Epidemiology (STROBE) statement for observational studies, Lancaster and Thabane’s guidance for non-randomized pilot and feasibility studies, and the Template for Intervention Description and Replication (TIDieR) checklist and guide for intervention description [20,21,22] (see Supplementary File S1). The study was not designed to estimate causal effectiveness. Its primary contribution is pre-confirmatory: to generate hypotheses and identify whether the available service-evaluation data structure can support a future longitudinal study.

### 2.2. Setting and Onboarding Pathway

The project was implemented at ASST Sette Laghi and involved two recruitment cohorts of international nurses entering the organization between late 2023 and mid-2024. The exact administrative denominator of unique nurses entering the pathway was not available in the anonymized analysis files and could not be reconstructed because stable participant identifiers were absent. The available survey data comprised 14 international-nurse response rows at T0 and T1 and 12 at T2; these counts represent responses rather than identifiable unique individuals. Organizational materials described the recruitment as involving Spanish-speaking/South American international nurses and indicated assignment to medical-area wards; however, the exact number of wards involved was not retained in the anonymized analysis files.

Before placement in the ward, the onboarding pathway included a pre-employment phase focused on Italian language preparation and professional alignment, followed by welcome training days covering the organization, ward roles, and information systems.

**Figure 1:**
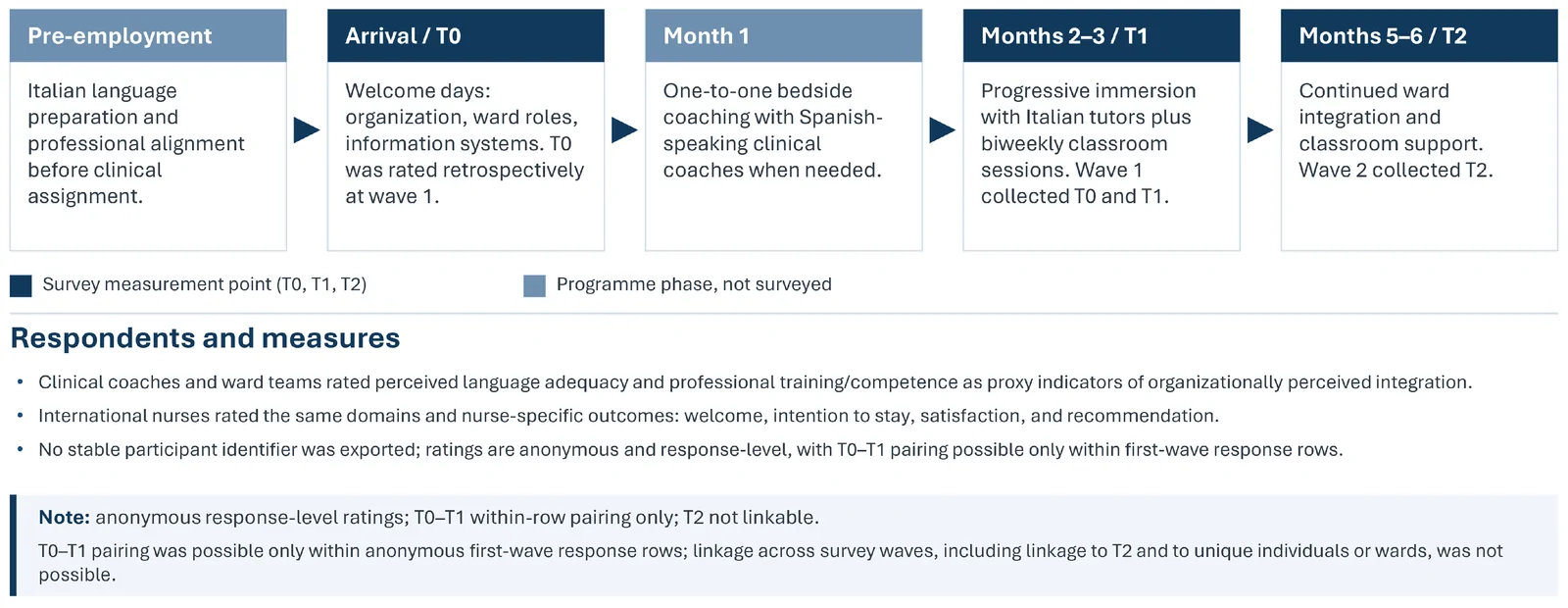
Onboarding and assessment timeline.

The workplace insertion phase combined one-to-one clinical coaching and parallel classroom education, as described in Figure 1.

During approximately the first month, clinical coaches who could communicate in Spanish were used when needed to make the transition gradual. Subsequently, experienced Italian nurses progressively replaced bilingual support to reinforce local-language immersion. Classroom education continued approximately every two weeks and addressed topics such as infection control, device management, and acute patient assessment. Coordinators and coaches used objective-based onboarding sheets to monitor progress and adjust support. These objective-based onboarding sheets were internal monitoring tools used by clinical coaches to structure the insertion pathway around progressive goals, including language use in clinical communication, familiarity with ward routines, technical and procedural skills, documentation, patient-safety practices, and progressive autonomy in daily nursing activities. The detailed sheets were not exported in the anonymized analysis files and were therefore not analyzed as separate instruments.

### 2.3. Participants and Respondent Groups

The target population comprised internationally educated nurses newly hired into ASST Sette Laghi within the two onboarding cohorts. Inclusion criteria were: nursing qualification obtained outside Italy; entry into ASST Sette Laghi between late 2023 and mid-2024; participation in the structured onboarding pathway; and assignment to medical-area wards. Ward-level identifiers and the exact number of medical-area wards involved were not retained in the anonymized analysis files.

Exclusion criteria for the nurse-specific dataset were: nursing education completed in Italy, prior practice in the Italian National Health Service before the observed onboarding pathway, non-completion of the pre-employment phase, and non-nursing support roles.

For proxy respondent groups, clinical coaches and ward-team members were eligible if they were directly involved in supervision of, or shared clinical practice with, the incoming international nurses during the observation period. Respondents without direct contact with the onboarding cohorts were not part of the survey.

To preserve anonymity under the organizational monitoring conditions, the analysis-ready survey exports did not include stable participant identifiers, sex, country of training, ward-level identifiers, language certification, or title-recognition status. This limits transferability and prevents individual longitudinal linkage. Available and unavailable baseline descriptors are summarized in Table 1.

### 2.4. Data Collection, Timepoints, and Questionnaire

Three respondent groups contributed anonymous questionnaire data: clinical coaches, ward team members, and newly hired international nurses. The primary questionnaire assessed perceived adequacy of language proficiency and professional training/competence. For clinical coaches and ward teams, these measures were proxy ratings of the nurses’ integration as perceived by the organization; for international nurses, they reflected self-perception. Because stakeholder groups differed in their relationship to the onboarding process, pooled analyses were interpreted as organizational response-level summaries rather than estimates of individual nurse change.

Questionnaires were administered in two waves. At approximately 3 months, respondents rated T0, referring to the arrival or early-days period, and T1, referring to the current 3-month status. T0 is therefore partly retrospective, subject to recall and contrast bias, and should not be interpreted as a prospective baseline. A subsequent administration at approximately 5–6 months captured T2. The instruments were purpose-designed, non-validated questionnaires originally developed for internal organizational monitoring. Items were developed pragmatically by the organizational project team to capture domains considered relevant for early onboarding and integration, including perceived language proficiency, professional training/competence, welcome, intention to stay, satisfaction, and willingness to recommend the experience. No formal expert-panel validation, Delphi process, content-validity index, or psychometric validation was conducted before data collection. Therefore, the items were treated as non-validated monitoring items, and the findings were interpreted as exploratory response-level indicators rather than validated measures of competence, integration, or retention. The complete available questionnaire structure is reported in Supplementary Table S1, including source fields, English analytic labels, target respondent groups, response format, scoring approach, and item/composite structure. The analysis-ready anonymized exports retained numeric five-point responses and operational item labels, but did not preserve the complete original verbal item wording or verbal anchor labels. Therefore, the thresholds of ≥3 and ≥4 were used only as pragmatic descriptive thresholds and should not be interpreted as validated cut-offs.

Because T0 and T1 were rated within the same first-wave questionnaire, these two ratings may be paired within response rows. However, the anonymized dataset did not include stable identifiers allowing linkage of the first-wave response rows to T2 or to unique nurses, respondents, or wards across survey waves.

Language proficiency and professional training/competence were analyzed as single five-point items in the primary dataset. In the nurse-specific dataset, welcome was captured through separate items for the organization, coordinator, ward team, and physicians; overall welcome was calculated as the arithmetic mean of the four component welcome items when available, with no imputation. In the main nurse-specific analytic subset used for welcome and retention-related analyses, all four welcome items were available for all 14 response rows. Intention to stay, satisfaction, and willingness to recommend the experience were analyzed as single items.

### 2.5. Outcomes and Feasibility Criteria

Primary exploratory outcomes were five-point Likert ratings of language proficiency and professional training/competence. The threshold of ≥3 was used pragmatically to indicate perceived adequacy on the five-point response scale, whereas ≥4 indicated a clearly favourable rating. Secondary outcomes among international nurses were overall welcome, intention to stay, satisfaction, and willingness to recommend the experience. In the nurse-specific dataset, welcome was assessed through four separate five-point items referring to welcome from the organization, nurse coordinator, ward team, and physicians. Overall welcome was calculated as the arithmetic mean of these four items and was used only as a pragmatic descriptive summary, not as a validated scale score. Internal consistency of the four welcome items was examined descriptively using Cronbach’s alpha. Because the sample was small and the welcome items referred to distinct organizational actors, individual welcome items were also presented separately and included in the correlation matrix.

Feasibility criteria were pragmatic: response yield across stakeholder groups and timepoints, completeness of key variables, clarity of denominators, availability of baseline descriptors, ability to link repeated observations, and usefulness of pilot-observed differences for planning a future study.

### 2.6. Statistical Analysis

The unit of analysis was the anonymous response row. For clinical-coach and ward-team data, each row represented one completed proxy-rating questionnaire from the relevant stakeholder group at a given conceptual timepoint. These rows should not be interpreted as verified unique staff members, unique international nurses, or cohort-level aggregate assessments. The anonymized analysis files did not include respondent identifiers, target-nurse identifiers, or ward identifiers. Therefore, multiple staff members may have assessed the same nurse, and the same respondent may have contributed more than one response, but this clustering structure could not be reconstructed. The primary analyses were descriptive. Because the data were ordinal, anonymous, and only partially linkable across conceptual timepoints, descriptive statistics emphasized medians, interquartile ranges, and threshold percentages. Means and standard deviations were retained as supportive descriptors because they were needed for approximate future sample-size planning. Analyses were conducted on available responses for each variable; no imputation was performed.

T0 and T1 ratings were collected within the same first-wave questionnaire and could therefore be paired within response rows when both values were available. However, stable identifiers were not available to link responses to unique nurses, respondents, wards, or T2 observations across survey waves. Accordingly, independent-sample comparisons between T0 and T1 were not interpreted as valid inferential evidence. Where T0 and T1 values were available within the same response row, paired T0–T1 changes were summarized as a sensitivity analysis. Comparisons involving T2 were interpreted descriptively because T2 could not be linked to the earlier wave through stable identifiers. The 127 T0–T1 pairs reported in Supplementary Table S3 were constructed directly from first-wave response rows in which both the retrospective T0 rating and the current T1 rating were available for the same domain. No participant identifiers, target-nurse identifiers, respondent identifiers, ward identifiers, or statistical matching procedure were used to create these pairs. Each paired difference therefore represents a within-row T1 minus T0 contrast from an anonymous first-wave questionnaire response.

Exploratory Kruskal–Wallis tests were retained only as screening descriptors for response-level differences across conceptual timepoints and were not used to support claims of causal or within-person change. For Kruskal–Wallis screening tests, effect size was reported as eta-squared based on H$\left(\eta_{H}^{2}\right)$, calculated as $\left(H-k+1\right)/\left(n-k\right)$, where H is the Kruskal–Wallis statistic, k is the number of groups, and *n* is the total number of observations. Pairwise Wilcoxon rank-sum comparisons were not interpreted in the main manuscript because of the anonymous, partially linkable data structure and the non-independence concerns for T0–T1 observations. Spearman correlations were used as the primary association summaries for secondary outcomes; Pearson correlations were retained only as sensitivity descriptors in the analysis report. For the nurse-specific secondary outcomes, false-discovery-rate adjustment was applied to the family of 28 pairwise Spearman correlation tests, using the Benjamini–Hochberg procedure implemented in R with p.adjust(method = “fdr”).

Future sample-size planning used observed mean differences and pooled standard deviations as approximate two-sample estimates for future unpaired studies at alpha = 0.05 and 80% or 90% power. Cohen’s d values in this context are planning descriptors only; they are not confirmatory effect-size estimates. A future linked longitudinal study should recalculate the sample size under the selected repeated-measures or mixed-effects model, using a prespecified primary outcome, a meaningful target difference, the expected within-person correlation, clustering structure, and attrition assumptions.

Analyses were conducted in R version 4.6.1 using the packages readxl 1.5.0, dplyr 1.2.1, tidyr 1.3.2, ggplot2 4.0.3, knitr 1.51, broom 1.0.13, psych 2.6.5, ggpubr 1.0.0, scales 1.4.0, and patchwork 1.3.2.

## 3. Results

### 3.1. Data Yield, Respondent Composition, and Feasibility Constraints

All findings are reported as response-level summaries, as shown in Table 2. Because no stable participant identifier was available, rows could not be linked across survey waves and should not be interpreted as individual longitudinal trajectories. T0 and T1 could be paired only within anonymous first-wave response rows when both ratings were available, whereas T2 could not be linked to the earlier wave. The primary aggregated dataset contained 410 response-level observations: 37 from clinical coaches, 333 from ward teams, and 40 from international nurses. The 410 observations represent response-level survey rows from multiple stakeholder groups, predominantly ward-team proxy ratings, rather than a sample of 410 nurses. Ward-team proxy ratings represented most of the pooled dataset (333/410 response rows, 81.2%). The respondent composition also varied across conceptual timepoints, with 127 response rows at T0, 127 at T1, and 156 at T2. Potential clustering within nurses, wards, or respondents could not be modelled because stable identifiers were not available.

The nurse-specific secondary datasets represented separate analytic subsets derived from the international-nurse questionnaires. Subset 1 contained 26 international-nurse response rows and subset 2 contained 14 international-nurse response rows. These rows should be interpreted as anonymous survey responses rather than as verified counts of unique nurses, because stable identifiers were not available. The 40 international-nurse rows reported in Table 2 refer to the primary response-level dataset for language proficiency and professional training/competence (14 at T0, 14 at T1, and 12 at T2). The nurse-specific secondary subsets were separate anonymized questionnaire exports used for welcome and retention-related analyses. Subset 1 contained 26 rows and subset 2 contained 14 rows; these subsets overlap with the international-nurse response data but should not be interpreted as additional participants or as a verified count of unique nurses. Because no stable identifiers or linkage keys were available, row-level correspondence between the primary dataset, the secondary subsets, and unique nurses could not be reconstructed.

The main feasibility limitation was the absence of stable pseudonymized identifiers. This prevented estimation of within-nurse change, linkage of repeated observations across waves, stakeholder-level clustering, ward-level clustering, and repeated-measures modelling. In addition, the exact administrative denominator of unique eligible nurses could not be reconstructed from the anonymized analysis files. The feasibility implications of these data-structure limitations are summarized against the pre-specified feasibility criteria in Table 3.

### 3.2. Stakeholder-Specific Descriptive Findings

Stakeholder-specific descriptive summaries are reported because self-ratings and proxy ratings represent related but non-equivalent constructs. Clinical coaches and ward-team members rated perceived integration from an organizational perspective, whereas international nurses provided self-ratings. In addition, the pooled dataset was compositionally unbalanced and dominated by ward-team proxy ratings, which accounted for 333 of 410 observations (81.2%). The respondent mix also changed across conceptual timepoints: clinical coaches contributed 11, 11, and 15 observations at T0, T1, and T2; ward-team members contributed 102, 102, and 129; and international nurses contributed 14, 14, and 12, respectively. Therefore, pooled T0-to-T2 patterns may partly reflect the changing composition of respondents across timepoints, in addition to differences in perceived integration. Detailed stakeholder-specific values are reported in Supplementary Table S2.

For language proficiency, coach and ward-team ratings showed more favourable response-level patterns at later timepoints. Among clinical coaches, the median rating moved from 2 (IQR 1–3) at T0 to 3 (IQR 3–4) at T2, and the proportion of ratings at or above 3 increased from 36.4% to 93.3%. Among ward-team members, the median moved from 2 (IQR 2–3) at T0 to 3 (IQR 3–4) at T2, and the proportion of ratings at or above 3 increased from 49.0% to 89.9%.

International nurses showed a different descriptive pattern. Their self-rated language proficiency was already high at retrospective T0, with a median of 4 (IQR 3–4), decreased to 3 (IQR 3–3) at T1, and returned to 4 (IQR 3–4) at T2. This divergence indicates that the pooled language pattern was largely driven by ward-team and coach proxy ratings and should not be interpreted as a uniform change across stakeholder perspectives.

For professional training/competence, coach and ward-team ratings again showed more favourable response-level patterns at later timepoints. Clinical coach ratings moved from a median of 3 (IQR 2–3) at T0 to 3 (IQR 3–4) at T2, while ward-team ratings moved from 2 (IQR 2–3) to 3 (IQR 3–4). International nurses rated this domain more favourably throughout, with median values of 4 at T0, T1, and T2. These findings reinforce the need to interpret stakeholder-specific results separately from pooled summaries. Pooled results are therefore presented only as organizational monitoring summaries, not as stakeholder-adjusted effects or estimates of individual nurse change.

### 3.3. Pooled Response-Level Exploratory Patterns

Pooled analyses are reported as organizational response-level summaries, not as estimates of individual nurse change (see Table 4). Across all stakeholder response rows, ratings showed progressively more favourable patterns at later conceptual timepoints.

**Figure 2:**
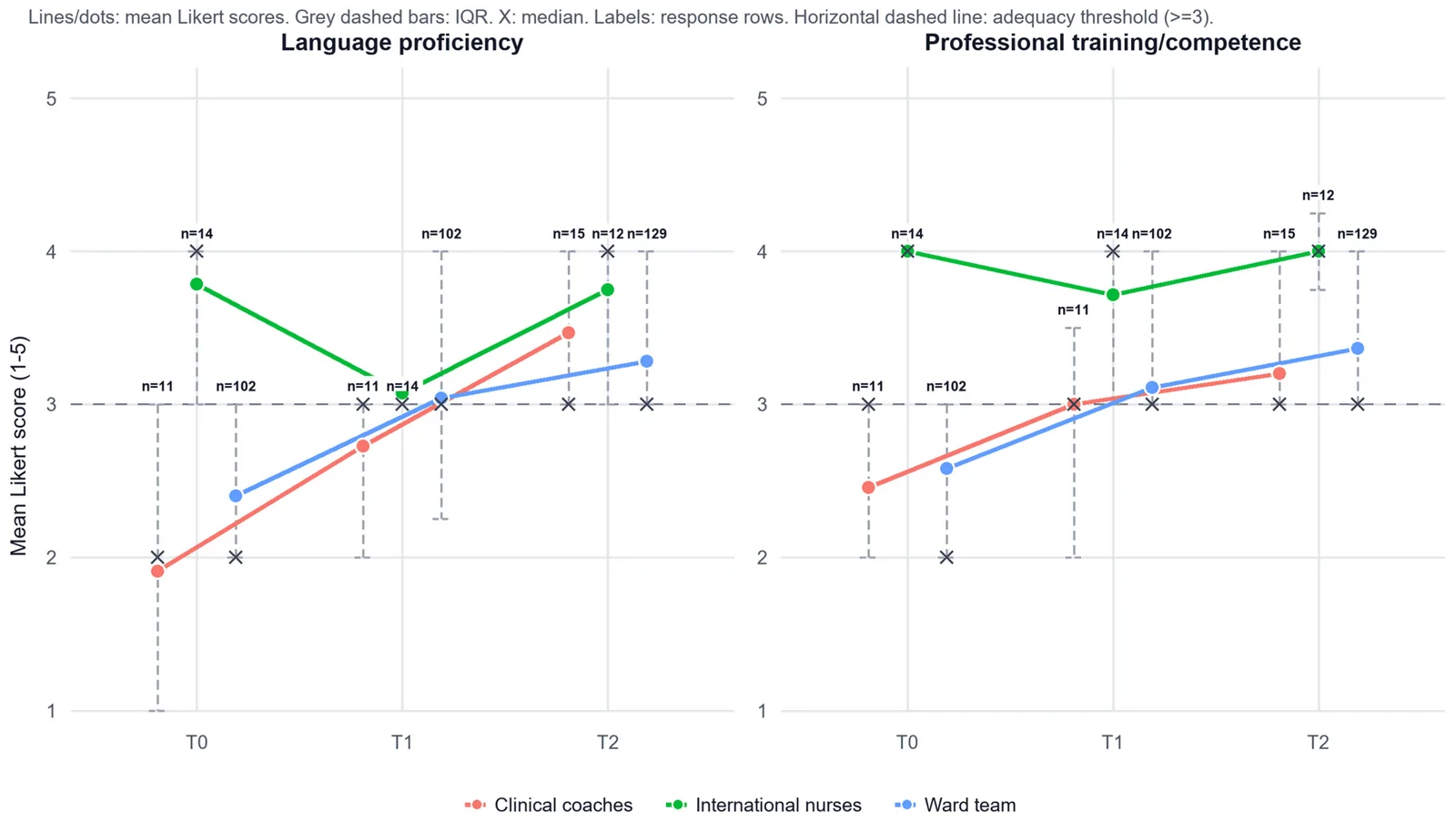
Stakeholder-specific response-level patterns in perceived language proficiency and professional training/competence across conceptual timepoints. Note: Coloured lines and dots show mean Likert scores; grey dashed vertical bars show the interquartile range; X markers indicate medians; labels report the number of anonymous response rows. The horizontal dashed line indicates the pragmatic adequacy threshold of ≥3.

For language proficiency, the median moved from 3 (IQR 2–3) at T0 to 3 (IQR 3–4) at T2, and the proportion of ratings at or above the pragmatic adequacy threshold increased from 53.5% to 91.0%. For professional training/competence, the median remained 3, but the IQR shifted from 2–4 to 3–4, and the corresponding proportion increased from 53.5% to 89.7% (see Figure 2).

Mean response-level ratings are reported only as supportive planning descriptors. Language proficiency moved from 2.51 at T0 to 3.02 at T1 and 3.33 at T2, while professional training/competence moved from 2.72 to 3.17 and 3.40. Because observations were anonymous, partly retrospective at T0, and potentially non-independent, these values should be interpreted descriptively.

**Figure 3:**
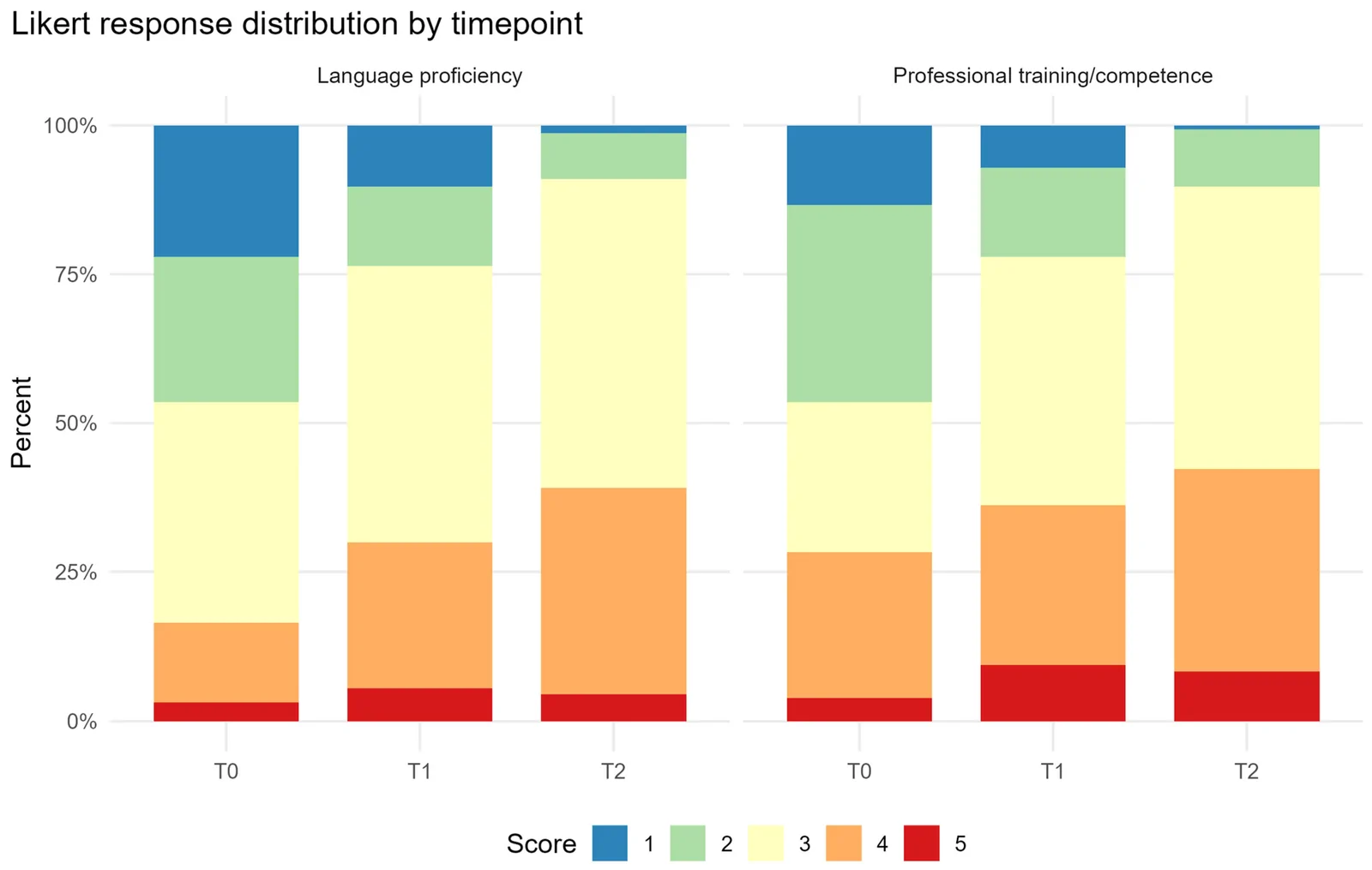
Likert response distribution by conceptual timepoint for language proficiency and professional training/competence.

The Likert response distributions showed a lower proportion of ratings in the least favourable categories at later conceptual timepoints and a higher concentration of ratings at 3 or above (see Figure 3). This pattern supports the descriptive interpretation of more favourable response-level ratings over time, while avoiding any assumption of individual longitudinal improvement. Exploratory Kruskal–Wallis screening tests indicated response-level differences across conceptual timepoints for language proficiency, H(2) = 46.21, *p* < 0.001, $\eta_{H}^{2}=0.109$, and professional training/competence, H(2) = 28.50, *p* < 0.001, $\eta_{H}^{2}=0.065$. These inferential results were treated only as descriptive screening information because observations were anonymous, T0 was partly retrospective, T0 and T1 were not fully independent by design, and stakeholder- or ward-level clustering could not be modelled. Accordingly, no post hoc inferential contrasts were used to support interpretation. A paired T0–T1 within-row sensitivity summary was examined for the first-wave response rows with both ratings available. For both primary domains, 127 response rows contained paired T0 and T1 ratings. Mean within-row ratings were higher at T1 than at T0 for language proficiency (mean delta = 0.50 points; 59 improved, 35 unchanged, and 33 worsened) and professional training/competence (mean delta = 0.44 points; 61 improved, 27 unchanged, and 39 worsened), although the median within-row change was 0 in both domains. This analysis was used only to assess compatibility with the descriptive response-level pattern and was not interpreted as confirmatory longitudinal evidence (Supplementary Table S3).

A paired T0–T1 within-row sensitivity summary was examined for the first-wave response rows with both ratings available. For both primary domains, 127 response rows contained paired T0 and T1 ratings. Mean within-row ratings were higher at T1 than at T0 for language proficiency (mean delta = 0.50 points; 59 improved, 35 unchanged, and 33 worsened) and professional training/competence (mean delta = 0.44 points; 61 improved, 27 unchanged, and 39 worsened), although the median within-row change was 0 in both domains. This analysis was used only to assess compatibility with the descriptive response-level pattern and was not interpreted as confirmatory longitudinal evidence (Supplementary Table S3).

### 3.4. Nurse-Specific Welcome and Retention-Related Outcomes

The four welcome items showed generally favourable but heterogeneous descriptive patterns among international nurses (n = 14; see Table 5). Median ratings were 4 for welcome from the organization, nurse coordinator, ward team, and physicians, although physician-related ratings showed greater dispersion. Internal consistency was modest (Cronbach’s alpha = 0.59), and this estimate was imprecise because of the small sample. Therefore, overall welcome was used only as a pragmatic summary indicator, while individual items were retained for descriptive summaries and the correlation matrix. 

**Figure 4:**
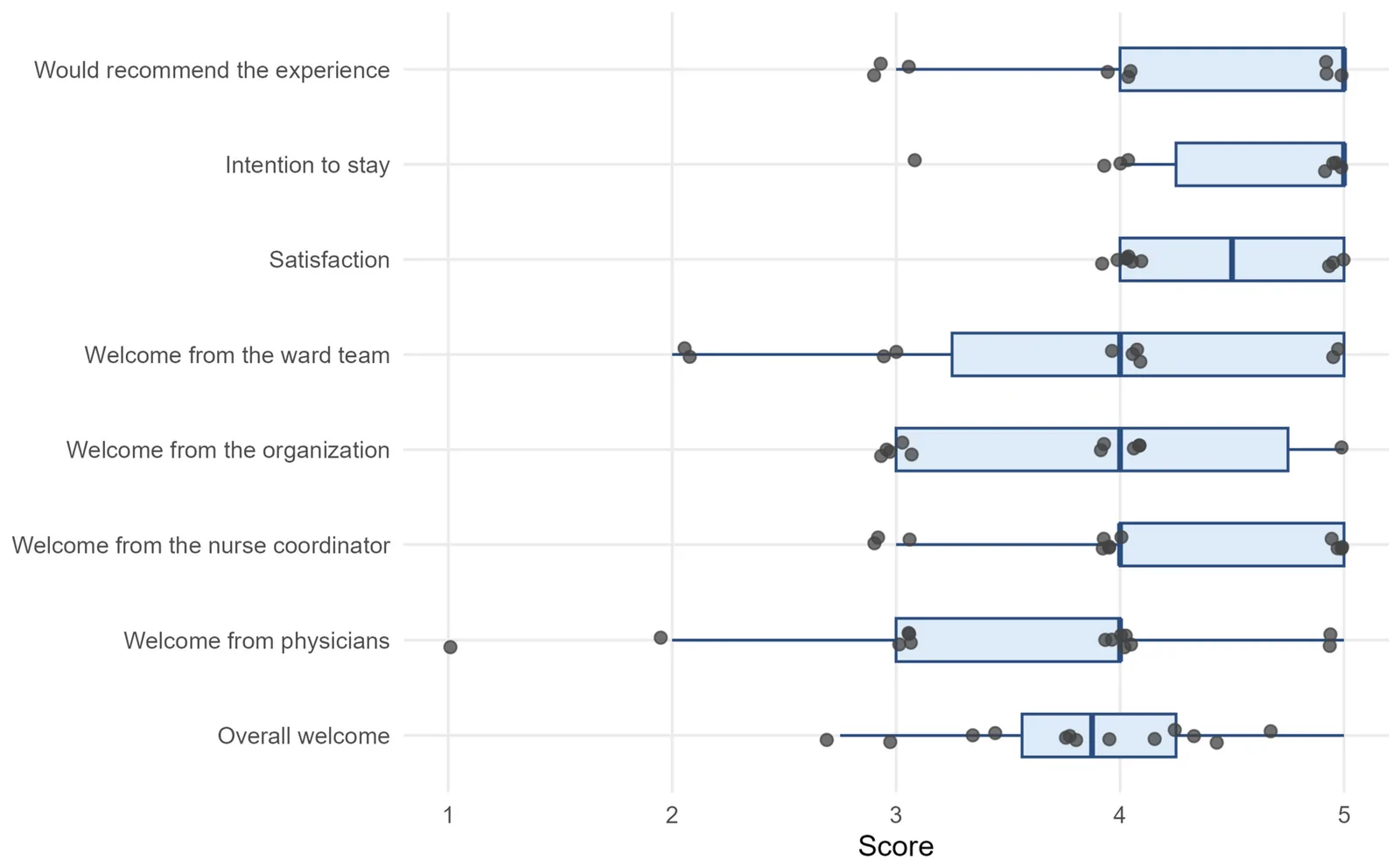
Secondary outcome distributions among international nurses.

Overall welcome had a median of 3.88 (IQR 3.56–4.25), intention to stay had a median of 5 (IQR 4.25–5), satisfaction had a median of 4.5 (IQR 4–5), and willingness to recommend the experience had a median of 5 (IQR 4–5) (see Figure 4).

Spearman correlations suggested that overall welcome was associated with intention to stay (rho = 0.73, approximate 95% CI 0.32 to 0.91, FDR *p* = 0.016) and willingness to recommend the experience (rho = 0.86, approximate 95% CI 0.60 to 0.95, FDR *p* = 0.002). The association with satisfaction was weaker and imprecise (rho = 0.23, approximate 95% CI −0.34 to 0.68, FDR-adjusted *p* = 0.565) (see Table 6).

These correlations should be interpreted as hypothesis-generating signals because of the small nurse-specific sample, the cross-sectional nature of the secondary dataset, the number of displayed correlations, and the possibility of common-method variance.

**Figure 5:**
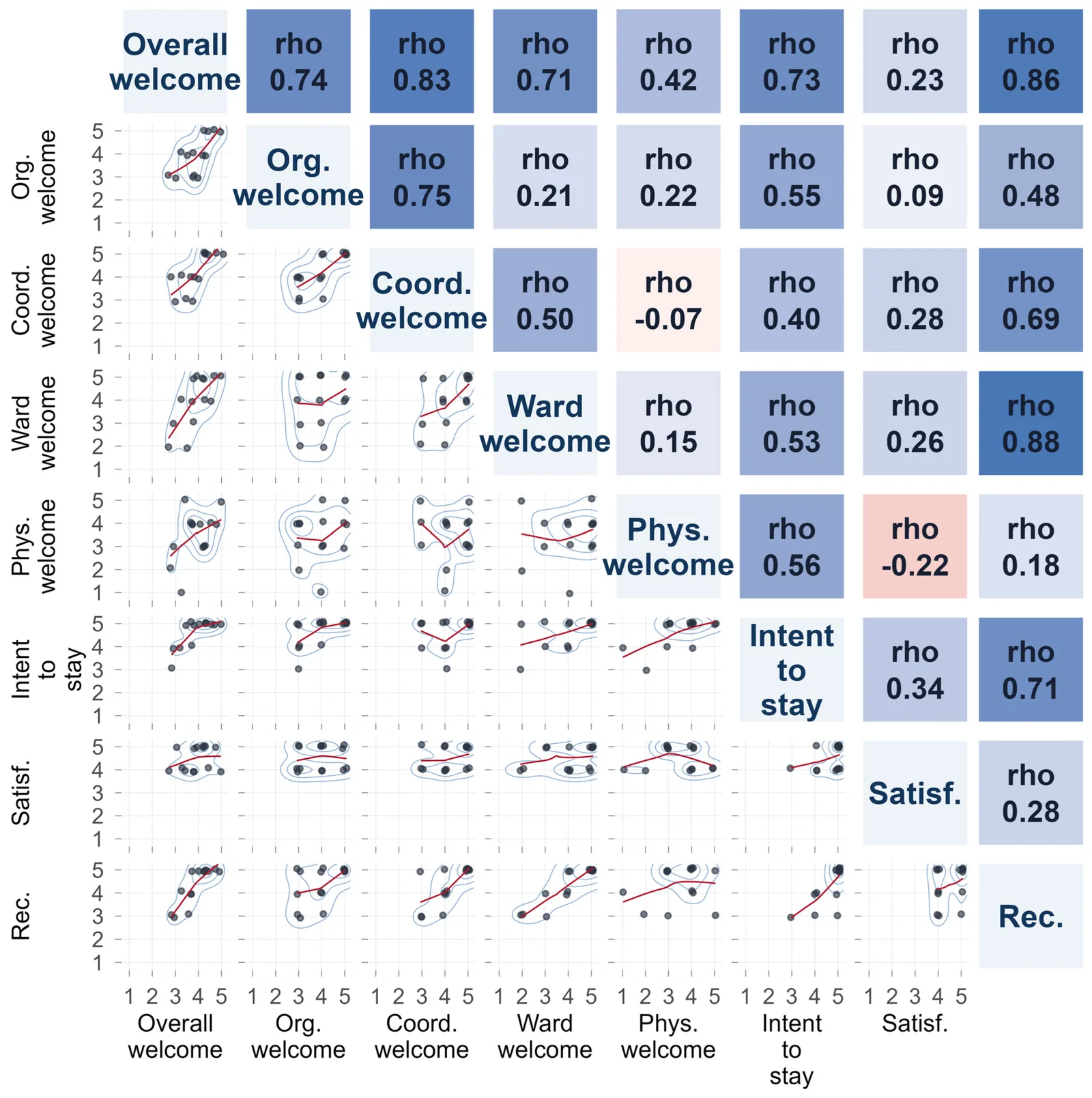
Spearman correlations and bivariate response patterns among international nurses. Note: The upper triangle displays Spearman rho coefficients for the 28 pairwise associations among eight welcome-related and retention-related variables; false-discovery-rate adjustment was computed across this full family of 28 tests. The lower triangle shows jittered Likert scatterplots with two-dimensional density contours and LOESS smoothers. Because of the small sample size and discrete response scale, the figure should be interpreted descriptively and as hypothesis-generating.

Figure 5 displays the full correlation structure among welcome-related dimensions and nurse-specific outcomes. Overall welcome showed positive correlations with willingness to recommend the experience (rho = 0.86, 95% CI 0.60 to 0.95; *p* < 0.001; FDR q = 0.002), coordinator welcome (rho = 0.83, 95% CI 0.53 to 0.94; *p* < 0.001; FDR q = 0.003), organizational welcome (rho = 0.74, 95% CI 0.35 to 0.91; *p* = 0.002; FDR q = 0.014), intention to stay (rho = 0.73, 95% CI 0.32 to 0.91; *p* = 0.003; FDR q = 0.016), and ward-team welcome (rho = 0.71, 95% CI 0.28 to 0.90; *p* = 0.005; FDR q = 0.018). Ward-team welcome was also strongly correlated with recommendation (rho = 0.88, 95% CI 0.65 to 0.96; *p* < 0.001; FDR q = 0.001), suggesting that the local clinical environment may be particularly relevant for how international nurses evaluated the onboarding experience. By contrast, satisfaction showed weaker and imprecise correlations with overall welcome (rho = 0.23, 95% CI −0.34 to 0.68; *p* = 0.424; FDR q = 0.565) and recommendation (rho = 0.28, 95% CI −0.30 to 0.70; *p* = 0.336; FDR q = 0.510), supporting the interpretation that satisfaction and welcome-related retention indicators may represent related but non-equivalent constructs. The lower triangle should be interpreted descriptively because of the small sample size, wide confidence intervals, and discrete response scale.

### 3.5. Feasibility Implications for Future Studies

The pilot data were used only to illustrate the possible order of magnitude of sample-size requirements under a simple planning scenario. These values should not be interpreted as definitive sample-size requirements, estimates of intervention effect, or protocol-ready calculations, because they are derived from anonymous, potentially non-independent response-level data based on non-validated ordinal monitoring items (Table 7). To reduce reliance on unstable pilot-observed effect sizes, Table 7 reports planning scenarios for the T0-to-T2 contrast using both the observed mean difference and the lower 95% confidence bound of that difference. For language proficiency, the observed T0-to-T2 difference was 0.82 points, with a lower-bound planning difference of 0.60 points; the corresponding approximate 80% power values were 21 and 36 observations per group/timepoint, respectively. For professional training/competence, the displayed T0-to-T2 difference was 0.68 points, with a lower-bound planning difference of 0.45 points; the corresponding approximate 80% power values were 32 and 71 observations per group/timepoint, respectively. These values are presented only as provisional planning parameters and should be recalculated in a future protocol using a prespecified primary outcome, a prospectively defined minimally important difference, expected within-person correlation, clustering structure, attrition assumptions, and the intended longitudinal or mixed-effects model.

## 4. Discussion

### 4.1. Principal Findings

This exploratory pilot study showed that a structured, language-aware onboarding pathway for international nurses can be evaluated through a multi-stakeholder response-level approach in a non-English-speaking Italian healthcare organization. The main contribution is not evidence of causal effectiveness, but the identification of promising response-level patterns and, more importantly, the data-infrastructure requirements needed for a future longitudinal evaluation.

The observed language and professional training patterns were generally more favourable at later conceptual timepoints for perceived language proficiency and professional training/competence. However, these findings should not be interpreted as individual improvement or as evidence that nurses reached an adequate level of practice after three months. The data were anonymous, T0 was partly retrospective, T0 and T1 may not have been fully independent by design, and stakeholder or ward-level clustering could not be modelled. Therefore, the findings are best interpreted as exploratory organizational monitoring signals that may inform future study design.

The findings are consistent with prior literature identifying language barriers, acculturation, professional role adaptation, organizational support, and transition shock as central issues for internationally educated and newly hired nurses [8,13,14]. The contribution of this pilot is more specific: it documents these issues in a non-English-speaking Italian healthcare context, evaluates a real organizational onboarding pathway rather than a purely descriptive barrier study, and tests whether routine multi-stakeholder monitoring data can support future evaluation [17,23,24].

### 4.2. Stakeholder-Specific Perceptions of Integration

A key finding of this pilot is that stakeholder-specific patterns were not identical. Coach and ward-team proxy ratings showed more favourable response-level patterns at later conceptual timepoints, whereas international nurses showed a different pattern, particularly for language proficiency. International nurses rated language proficiency relatively highly at retrospective T0, lower at T1, and again more favourably at T2. This divergence suggests that pooled estimates may conceal meaningful differences between self-perceived and organizationally perceived integration [25,26].

This finding is methodologically important because nurse self-ratings and coach or ward-team proxy ratings do not measure the same construct. Self-ratings reflect the nurse’s own perception of language, competence, and adaptation, whereas proxy ratings reflect how integration is perceived by the receiving organization. Both perspectives are relevant, but they should not be treated as interchangeable. In future studies, stakeholder-specific outcomes should be prespecified and analyzed separately, with multilevel or mixed-effects models used where repeated measures and clustering could be identified.

Substantively, the divergence between international nurse self-ratings and proxy ratings may also reflect different frames of reference. International nurses may initially evaluate their ability in relation to prior professional experience, whereas ward teams and coaches may evaluate communication and competence in relation to the demands of the Italian clinical environment. As nurses become more exposed to local expectations, self-assessment may become more calibrated [27]. This interpretation remains exploratory, but it reinforces the need to include multiple perspectives when evaluating onboarding.

### 4.3. Welcome and Retention-Related Outcomes

The association between perceived welcome and retention-related outcomes represents one of the most relevant hypothesis-generating findings of this pilot. Welcome is a potentially modifiable organizational factor and may capture aspects of belonging, recognition, and relatedness that are particularly salient during early professional transition [2,3,24,25]. Its stronger association with intention to stay and willingness to recommend the experience than with satisfaction suggests that welcome and general satisfaction may not be interchangeable constructs.

These findings align with recent literature emphasizing organizational support, relatedness, acculturation, and retention as central dimensions of international nurse integration [28]. However, the correlation findings should be interpreted cautiously. The nurse-specific sample was small, the measures were self-reported and cross-sectional, and the same questionnaire captured welcome, intention to stay, satisfaction, and recommendation. Common method variance or a general positive response tendency may therefore partly explain the observed associations. Future longitudinal studies should assess whether perceived welcome prospectively predicts retention-related outcomes after adjustment for baseline characteristics, ward context, language proficiency, and professional adaptation.

### 4.4. Feasibility Lessons for Future Longitudinal Evaluation

The feasibility findings are central to the interpretation of this pilot. The study demonstrated that routine multi-stakeholder monitoring data can be collected and analyzed, but also showed that the current data structure is not sufficient for confirmatory longitudinal evaluation. Although T0 and T1 could be paired within anonymous response rows when both values were present, the lack of stable identifiers prevented linkage to T2, linkage to unique nurses, respondents, or wards, and repeated-measures modelling across survey waves.

The pilot also highlighted the need for clearer denominators and more systematic feasibility reporting. Future studies should prospectively record the number of eligible, invited, participating, and completing international nurses, as well as response rates by stakeholder group and timepoint [29,30]. Item-level missingness, stakeholder composition, intervention completion, ward exposure, and baseline descriptors should also be defined before data collection. In this sense, the pilot not only generated preliminary outcome signals; it clarified the operational requirements for a more rigorous future evaluation [31,32].

The sample-size values reported in this study should therefore be considered provisional planning aids only. They were derived from pilot-observed response-level variability and should not be interpreted as stable estimates of intervention effect. A future confirmatory study should base sample-size calculation on a prespecified primary outcome, a clinically or organizationally meaningful difference, expected within-person correlation, clustering structure, attrition, and the intended longitudinal or mixed-effects model.

### 4.5. Limitations

This study has several limitations that directly affect interpretation and generalizability. First, although T0 and T1 could be paired when both ratings were available within the same anonymous response record, the absence of stable pseudonymized identifiers prevented linkage to T2, individual nurses, or wards. Consequently, within-nurse change and repeated-measures or clustered analyses across survey waves could not be estimated. Second, T0 was partly retrospective rather than a prospective baseline; ratings of arrival and the early days may therefore have been affected by recall bias, contrast with later experiences, or response-shift effects. Third, the independence of observations could not be guaranteed. Responses may have been clustered within nurses, ward teams, wards, or respondents, but these structures were not available in the anonymized dataset. Therefore, inferential analyses were treated only as exploratory screening tools. Fourth, the pooled analyses combined nurse self-ratings with coach and ward-team proxy ratings. These perspectives are related, but not equivalent, and pooled summaries were dominated by ward-team responses. Stakeholder-specific findings are therefore essential for interpretation. Fifth, the questionnaires were purpose-designed for internal organizational monitoring and were not validated research instruments. The items should be interpreted as exploratory monitoring indicators rather than validated measures of language proficiency, professional competence, welcome, or retention. Sixth, the nurse-specific correlation analysis was based on a small cross-sectional sample. Confidence intervals were wide, and the findings may reflect common method variance or a general positive response tendency. Finally, the study was conducted in a single Italian healthcare organization. Transferability is limited by the local organizational context, the specific onboarding pathway, incomplete denominators, and the lack of linkable baseline descriptors.

### 4.6. Implications for Practice and Research

Practical implications should be framed as feasibility-informed rather than effectiveness-confirming. The pilot suggests that a sequenced, language-aware pathway with pre-employment preparation, welcome days, transitional bilingual coaching, later immersion with Italian tutors, and classroom support can generate analyzable multi-stakeholder data. Healthcare organizations considering international recruitment should treat onboarding and welcome as workforce-retention support, while also building the measurement systems needed to evaluate these efforts rigorously [31,32]. For future research, the next step should be a prospective longitudinal evaluation with stable, pseudonymized identifiers; stakeholder-specific outcomes; validated or formally adapted instruments; objective language and professional indicators where possible; and prespecified feasibility-, implementation-, and effectiveness-related endpoints. The design should allow paired analyses, modelling of clustering, and estimation of within-nurse change [33]. Only with this stronger data infrastructure will it be possible to determine whether structured onboarding is associated with sustained improvements in integration and retention-related outcomes [34].

## 5. Conclusions

This study, concerning one of the first experiences of a structured onboarding pathway for international nurses in an Italian healthcare organization, generated promising response-level signals and clarified both the feasibility and limitations of a multi-stakeholder evaluation approach. This pilot does not demonstrate individual improvement or causal effectiveness; rather, it supports hypotheses about language-aware onboarding, organizationally perceived integration, and welcome-related retention outcomes. Future confirmatory research will require a stronger prospective data infrastructure, including stable identifiers, validated measures, and longitudinal analytic models.

## Figures and Tables

**Table 1:** Baseline and transferability descriptors available from the anonymized project materials.

Descriptor	Availability	Available Summary	Implications for Study Reporting and Interpretation
Target recruitment cohorts	Partly available	Two international-nurse recruitment cohorts entered the programme between late 2023 and mid-2024. The exact administrative denominator of unique nurses was not available in the anonymized analysis files.	Survey counts represent response-level observations and cannot be converted into unique-participant denominators without stable identifiers.
Region/language background	Partly available	Organizational materials described the recruitment as involving Spanish-speaking international nurses from South America. However, country-specific counts were not retained in the anonymized analysis files and could not be linked to the analytic response rows.	Country-level counts are not reported as analytic denominators. Future studies should report aggregate country and language descriptors using privacy-preserving categories.
Age and sex	Partly available	Age categories were available in the survey responses but could not be linked to unique nurses across waves. Sex was not available in the analysis-ready dataset.	These variables should be recovered only as aggregate administrative descriptors, where permitted.
Pre-migration nursing experience	Partly available	Categories of previous nursing experience were present in the nurse-specific survey sheets but could not be linked across waves.	These data should be recovered only as aggregate administrative descriptors, where permitted.
Italian language certification	Not available	No objective Common European Framework of Reference for Languages (CEFR/QCER) level was available in the analysis-ready dataset.	Future studies should prospectively collect certified language-proficiency levels at programme entry.
Title recognition status	Available	Organizational information indicated that the nurses held temporary recognition of their academic qualifications.	This information should be reported only as an aggregate cohort descriptor because it could not be linked to individual survey responses.
Ward assignment	Partly available	Organizational materials indicated assignment to medical-area wards, but ward-level identifiers were not exported to the analysis-ready dataset.	Ward-level identifiers were omitted to reduce the risk of indirect identification. Future studies should use privacy-preserving ward or unit codes when clustering is to be modelled.

Note: CEFR = Common European Framework of Reference for Languages: Learning, teaching, assessment.

**Table 2:** Response-level data yielded by respondent group and conceptual timepoint.

Respondent Group	T0	T1	T2	Total Response Rows
Clinical coaches	11	11	15	37
Ward team	102	102	129	333
International nurses	14	14	12	40
Total response-level observations	127	127	156	410

**Table 3:** Feasibility outcomes against pre-specified feasibility criteria.

Pre-Specified Feasibility Criterion	Observed Result	Interpretation for This Pilot	Requirement for Future Study
Response yield across stakeholder groups and timepoints	A total of 410 response-level observations were available: 37 from clinical coaches, 333 from ward-team members, and 40 from international nurses.	Multi-stakeholder data collection was feasible, although the dataset was predominantly composed of ward-team proxy ratings.	Predefine target denominators and expected response rates for each stakeholder group.
Denominator clarity	The exact administrative denominator of unique eligible nurses could not be reconstructed from the anonymized analysis files.	Uncertainty regarding the denominator limited the interpretation of response rates and external transferability.	Prospectively record the numbers of eligible, invited, participating, and completing nurses.
Stable identifiers and linkage	No stable pseudonymized participant identifier was available.	Individual responses could not be linked across survey waves or analyzed as longitudinal trajectories.	Implement stable pseudonymized participant identifiers across all data collection timepoints.
Missingness and item completeness	Primary variables were complete for 410/410 response rows: language proficiency (410/410) and professional training/competence (410/410). In the nurse-specific subset used for the main welcome/retention analyses, all reported secondary variables were complete across all 14 rows. In nurse-specific subset 1, individual welcome items and retention-related items were complete for all 26 rows, whereas the exported overall welcome composite was available for only 14 of the 26 rows. No imputation was performed.	Available-case analysis was feasible, but item-level missingness should be reported more systematically.	Predefine thresholds for acceptable item completeness and a missing-data management strategy.
Stakeholder composition	Ward-team ratings represented 333/410 (81.2%) of all response rows, and respondent composition varied across timepoints.	Overall estimates were strongly influenced by ward-team proxy ratings.	Analyze stakeholder groups separately and account for stakeholder composition in statistical models where appropriate.
Baseline and transferability descriptors	Several participant descriptors could not be linked at the individual level.	Assessment of transferability was limited by the absence of linkable baseline information.	Collect both aggregate and linkable baseline descriptors, consistent with privacy regulations.
Questionnaire reproducibility	The questionnaires were purpose-designed for this project and had not undergone formal validation.	The pilot was suitable for monitoring implementation indicators but not for measuring validated constructs.	Provide the complete survey instruments and consider using validated or formally adapted questionnaires in future studies.
Future longitudinal analysis	Repeated-measures analyses could not be performed because individual responses could not be linked over time.	The pilot successfully identified important limitations of the current monitoring data infrastructure.	Adopt prospective longitudinal or mixed-effects designs incorporating participant identifiers, clustering variables, and prespecified outcomes.
Intervention completion	Intervention-completion status and completion of individual onboarding components were not retained in the anonymized analysis files.	This criterion was unassessable in the present dataset and should be considered an unmet data-capture requirement for confirmatory evaluation.	Prospectively record start, completion, discontinuation, and dose/exposure for each onboarding component.

**Table 4:** Primary exploratory outcomes by timepoint.

Domain	Timepoint	n	Median (IQR)	Mean (SD)	≥3	≥4
Language proficiency	T0	127	3 (2–3)	2.51 (1.07)	53.5%	16.5%
Language proficiency	T1	127	3 (3–4)	3.02 (1.01)	76.4%	29.9%
Language proficiency	T2	156	3 (3–4)	3.33 (0.74)	91.0%	39.1%
Professional training/competence	T0	127	3 (2–4)	2.72 (1.1)	53.5%	28.3%
Professional training/competence	T1	127	3 (3–4)	3.17 (1.03)	78.0%	36.2%
Professional training/competence	T2	156	3 (3–4)	3.4 (0.8)	89.7%	42.3%

Note: Median and IQR are emphasized because variables are ordinal; means and SDs are supportive descriptors.

**Table 5:** Secondary outcomes among international nurses.

Outcome	n	Median (IQR)	Mean (SD)	≥4
Overall welcome	14	3.88 (3.56–4.25)	3.91 (0.65)	50.0%
Intention to stay	14	5 (4.25–5)	4.64 (0.63)	92.9%
Satisfaction	14	4.5 (4–5)	4.5 (0.52)	100.0%
Would recommend the experience	14	5 (4–5)	4.36 (0.84)	78.6%

**Table 6:** Welcome-related Spearman correlations among international nurses.

Association	n	Spearman rho	Approx. 95% CI	FDR *p*
Overall welcome with Intention to stay	14	0.73	0.32 to 0.91	0.016
Overall welcome with Satisfaction	14	0.23	−0.34 to 0.68	0.565
Overall welcome with Would recommend experience	14	0.86	0.60 to 0.95	0.002

Note: Confidence intervals are approximate Fisher z intervals and are reported to show imprecision in this small exploratory sample. FDR *p* = false discovery rate-adjusted *p*-value.

**Table 7:** Future-study sample-size planning scenarios based on observed and lower-bound T0-to-T2 response-level differences.

Domain	Planning Contrast	Planning Scenario	Mean Difference Used	Approx. 95% CI for Observed Difference	Cohen’s d Used	n/Group (80% Power)	n/Group (90% Power)
Language proficiency	T0 to T2	Observed pilot difference	0.82	0.60 to 1.04	0.91	21	27
Language proficiency	T0 to T2	Lower-bound scenario	0.60	0.60 to 1.04	0.66	36	48
Professional training/competence	T0 to T2	Observed pilot difference	0.68	0.45 to 0.90	0.71	32	43
Professional training/competence	T0 to T2	Lower-bound scenario	0.45	0.45 to 0.90	0.47	71	95

Note: Values are illustrative planning scenarios and are not estimates of intervention effectiveness or definitive sample-size requirements. The lower-bound scenario uses the lower 95% confidence bound of the observed T0-to-T2 response-level mean difference. Values are rounded for presentation; mean differences are displayed to two decimal places for consistency with the rounded means in Table 4, and small discrepancies may occur when calculations are based on unrounded values. A future confirmatory study should define a minimally important difference prospectively and recalculate the sample size under the selected longitudinal, repeated-measures, or clustered model, accounting for expected within-person correlation, clustering, and attrition.

## Data Availability

The anonymized response-level dataset is not publicly deposited because the institutional authorization does not allow prospective open-repository sharing. The dataset may be made available by the corresponding author upon reasonable and scientifically motivated request, subject to institutional approval and applicable privacy restrictions.

## Supplementary Materials

The following supporting information can be downloaded at: https://www.mdpi.com/article/10.3390/healthcare14162557/s1, Supplementary File S1. STROBE and TIDieR reporting alignment; Table S1: Questionnaire items, response anchors, scoring approach, and item/composite structure; Table S2: Stakeholder-specific primary descriptive summaries by domain and conceptual timepoint; Table S3. Paired T0–T1 sensitivity summary for primary response-level outcome.

## Abbreviations

The following abbreviations are used in this manuscript:

ASST	Azienda Socio Sanitaria Territoriale
CEFR	Common European Framework of Reference for Languages
CI	Confidence interval
FDR	False discovery rate
IQR	Interquartile range
LOESS	Locally estimated scatterplot smoothing
SD	Standard deviation
T0	Timepoint 0
T1	Timepoint 1
T2	Timepoint 2
TIDieR	Template for Intervention Description and Replication
