# Supplementary material for: Structured Onboarding of International Nurses in an Italian Healthcare Organization: An Exploratory Multi-Stakeholder Pilot and Feasibility Observational Study
Source: Healthcare (Basel). 2026 Aug 15;14(16):2557. doi: 10.3390/healthcare14162557 (PMC13512028; doi:10.3390/healthcare14162557)
Supplement: Supplementary file 1 [file healthcare-14-02557-s001.zip › Supplementary_FileS1.pdf]

Supplementary File S1. STROBE and TIDieR reporting alignment

This author-generated supplement maps the revised manuscript to STROBE domains for observational reporting and TIDieR domains for intervention description. It is an alignment aid and does not reproduce the full official checklists. The manuscript remains an exploratory response-level pilot and feasibility evaluation, not a confirmatory effectiveness study.

Panel A. STROBE reporting alignment

Guideline	Item / domain	Revised manuscript location	Reported or revised content	Notes / residual limitations
STROBE	1. Title and abstract	Title; Abstract	Title identifies the study as an exploratory multi-stakeholder pilot and feasibility evaluation; abstract now emphasizes anonymous response-level observations, medians/IQRs, threshold percentages, and exploratory correlations.	Means are not the leading abstract summary.
STROBE	2. Background / rationale	Introduction	Rationale reorganized to move from the Italian context to the wider international nurse-migration literature and to specify the non-English-speaking setting gap.	The evidence gap is presented as empirical and feasibility-oriented.
STROBE	3. Objectives	End of Introduction	Aims specify response-level patterns, welcome and retention-related outcomes, feasibility of routine data structure, and future study planning.	Causal effectiveness is not stated as an aim.
STROBE	4. Study design	Methods 2.1	Design is described as an exploratory, unpaired, response-level observational pilot and feasibility evaluation.	The manuscript distinguishes conceptual timepoints from verified longitudinal follow-up.
STROBE	5. Setting	Methods 2.2; Figure 1; Table 1	Setting, two recruitment cohorts, Italian healthcare organization, medical-area ward context, and onboarding sequence are described.	Some baseline administrative descriptors were unavailable in the anonymized files.
STROBE	6. Participants	Methods 2.3; Results 3.1; Tables 2 and 3	Respondent groups are clinical coaches, ward teams, and international nurses; response-row denominators are reported by group and timepoint.	Response rows are not interpreted as verified counts of unique participants.
STROBE	7. Variables	Methods 2.4; Supplementary Table S1	Primary monitoring items, nurse-specific welcome and retention-related variables, thresholds, and composite structure are specified.	Questionnaires were non-validated monitoring instruments.
STROBE	8. Data sources / measurement	Methods 2.3-2.4; Supplementary Table S1	Questionnaire source fields, analytic labels, response coding, item/composite structure, and missing original verbal anchors are documented.	Complete original verbal wording was not preserved in the analysis-ready exports.
STROBE	9. Bias	Methods 2.3; Methods 2.6; Discussion limitations	Key bias risks are reported: retrospective T0, anonymous unlinked rows, non-validated items, self-ratings mixed with proxy ratings, common-method variance, and clustering not modelled.	These limitations directly constrain causal and longitudinal interpretation.
STROBE	10. Study size	Methods 2.6; Results 3.1; Table 3; Table 7	Pilot sample size is described as available response-level data; future planning scenarios are reported separately.	The pilot was not powered to estimate intervention effects.
STROBE	11. Quantitative variables	Methods 2.4; Methods 2.6	Ordinal variables are summarized using medians, IQRs, threshold percentages, and supportive means/SDs for planning.	Thresholds >=3 and >=4 are pragmatic and not validated cut-offs.
STROBE	12. Statistical methods	Methods 2.6	Available-response analysis, no imputation, exploratory Kruskal-Wallis screening, Spearman correlations, FDR correction for 28 Spearman tests, and future planning methods are specified.	Inferential p values are screening descriptors only.
STROBE	13. Participants / response flow	Results 3.1; Table 2; Table 3	Response-level yield is reported by respondent group and conceptual timepoint, including nurse-specific subsets.	Unique eligible-nurse denominators could not be reconstructed.
STROBE	14. Descriptive data	Table 1; Table 2; Supplementary Table S2	Available demographic/contextual descriptors and stakeholder-specific descriptive summaries are reported.	Country-level counts and several baseline descriptors are not reported as analytic denominators.
STROBE	15. Outcome data	Results 3.2-3.4; Tables 4-6; Figures 2-5	Primary and secondary outcome summaries are reported using medians/IQRs, threshold percentages, and correlation summaries.	Outcomes are interpreted as response-level monitoring indicators.
STROBE	16. Main results	Results 3.2-3.5	Stakeholder-specific results are presented before pooled summaries; pooled results are framed as organizational monitoring summaries.	No stakeholder-adjusted effects or within-nurse changes are claimed.
STROBE	17. Other analyses	Figure 5; Supplementary Table S3;	Correlation matrix, paired best-pairing sensitivity summary, and sample-size	Sensitivity analyses are not confirmatory longitudinal

Guideline	Item / domain	Revised manuscript location	Reported or revised content	Notes / residual limitations
		Table 7	planning scenarios are reported.	analyses.
STROBE	18. Key results	Discussion 4.1	Discussion opens with the main feasibility contribution and data-infrastructure requirements for future longitudinal evaluation.	Effectiveness language was reduced.
STROBE	19. Limitations	Discussion limitations subsection	Limitations are organized around linkage/identifiers, retrospective T0, non-validated tools, proxy/self-rating pooling, small nurse-specific subset, and single-center transferability.	Limitations are linked to interpretation and generalizability.
STROBE	20. Interpretation	Discussion	Findings are interpreted as hypothesis-generating pilot signals and feasibility lessons rather than causal evidence.	Common-method variance and response tendency are acknowledged for correlations.
STROBE	21. Generalisability	Table 1; Discussion limitations / implications	Transferability is discussed with emphasis on the Italian non-English-speaking setting and unavailable baseline descriptors.	Future studies should collect aggregate descriptors prospectively.
STROBE	22. Funding / role of funder	Funding statement	Funding/role-of-funder information is reported in the manuscript statement section according to journal requirements.	If no external funding was received, this should remain explicitly stated.

Panel B. TIDieR intervention-description alignment

Guideline	Item / domain	Revised manuscript location	Reported or revised content	Notes / residual limitations
TIDieR	1. Brief name	Title; Methods 2.2; Figure 1	The intervention is described as a structured, language-aware onboarding pathway for international nurses.	The term is used consistently after revision.
TIDieR	2. Why	Introduction; Methods 2.2	Rationale includes workforce shortage, international nurse integration, language/professional adaptation, welcome, and retention-sensitive onboarding.	The rationale is framed as pilot/feasibility, not causal effectiveness.
TIDieR	3. What materials	Methods 2.2; Figure 1; Supplementary Table S1	Reported materials include organizational welcome days, language/professional preparation, monitoring questionnaires, and classroom support topics where available.	Detailed training handouts or teaching materials were not exported for analysis.
TIDieR	4. What procedures	Methods 2.2; Figure 1	Procedures include pre-employment preparation, welcome days, one-to-one clinical coaching, gradual transition from bilingual to Italian-speaking support, ward immersion, and classroom education.	Procedure details are reported as organizational pathway components.
TIDieR	5. Who provided	Methods 2.2; Figure 1	Clinical coaches, ward teams, experienced Italian nurses/tutors, and educators contributed to the onboarding pathway.	Provider-level identifiers and formal fidelity logs were not available.
TIDieR	6. How	Methods 2.2; Figure 1	Delivery modes included pre-employment preparation, in-person welcome/training days, ward-based one-to-one coaching, classroom sessions, and workplace immersion.	Remote versus in-person details are reported only where available.
TIDieR	7. Where	Methods 2.2; Table 1	The pathway was implemented in ASST Sette Laghi, Italy, with nurses assigned to medical-area wards.	Ward identifiers were not exported to preserve anonymity.
TIDieR	8. When and how much	Methods 2.2; Figure 1	Timing includes pre-employment preparation, welcome days, approximately one month of bilingual coaching when needed, months 2-3 immersion, T1 at about 3 months, and T2 at about 5-6 months.	Exact dose received by each nurse could not be linked without stable identifiers.
TIDieR	9. Tailoring	Methods 2.2; Figure 1	Tailoring included use of Spanish-speaking clinical coaching when needed and progressive transition to Italian-speaking tutors/support.	Tailoring was described organizationally, not measured at individual level.
TIDieR	10. Modifications	Methods 2.2; Discussion limitations	No formal, time-stamped intervention modifications were available in the analysis-ready files.	Future studies should document protocol changes and implementation adaptations prospectively.
TIDieR	11. How well planned	Methods 2.6; Table 3	Feasibility criteria and monitoring structure are reported; data-infrastructure requirements for future fidelity and longitudinal evaluation are specified.	No formal fidelity checklist was used in this pilot monitoring dataset.
TIDieR	12. How well actual	Results 3.1; Table 3; Discussion	The pathway generated analyzable multi-stakeholder monitoring data and	Actual fidelity/adherence at individual level could not

Guideline	Item / domain	Revised manuscript location	Reported or revised content	Notes / residual limitations
			identified missing infrastructure for confirmatory evaluation.	be estimated.

Abbreviations: FDR, false-discovery rate; IQR, interquartile range; STROBE, Strengthening the Reporting of Observational Studies in Epidemiology; TIDieR, Template for Intervention Description and Replication.
