# Supplementary material for: Structured Onboarding of International Nurses in an Italian Healthcare Organization: An Exploratory Multi-Stakeholder Pilot and Feasibility Observational Study
Source: Healthcare (Basel). 2026 Aug 15;14(16):2557. doi: 10.3390/healthcare14162557 (PMC13512028; doi:10.3390/healthcare14162557)
Supplement: Supplementary file 1 [file healthcare-14-02557-s001.zip › Supplementary_Table_S1_questionnaire_items.pdf]

Supplementary Table S1. Questionnaire items, response anchors, scoring approach, and item/composite structure

The instruments were purpose-designed, non-validated monitoring questionnaires developed for internal organizational use. Exact original verbal item wording and anchor labels were not preserved in the analysis-ready Excel exports; therefore, this table reports source fields, English analytic labels, item/composite structure, and the scoring approach used for the manuscript analyses.

Panel	Dataset / questionnaire	Respondent group	Source field(s)	English analytic label	Item / composite structure	Response anchors / coding	Scoring and analysis use
A. Contextual fields	Primary multi-stakeholder monitoring questionnaire	Clinical coaches; ward team; international nurses	Campione	Respondent group	Categorical descriptor	Coach; Equipe; Neoax/Neoassunti, recoded as clinical coaches, ward team, and international nurses.	Used for stakeholder-specific descriptive summaries and to interpret pooled response-level analyses.
A. Contextual fields	Primary multi-stakeholder monitoring questionnaire	Clinical coaches; ward team; international nurses	Gruppo	Recruitment cohort	Categorical descriptor	Cohort coded as 1 or 2 in the analysis-ready export.	Used descriptively; not interpreted as an intervention group comparison.
A. Contextual fields	Primary multi-stakeholder monitoring questionnaire	Clinical coaches; ward team; international nurses	TEMPO	Conceptual timepoint	Assessment timing descriptor	0 = T0 arrival/early-days rating; 1 = T1 approximately 3-month rating; 2 = T2 approximately 5-6-month rating.	Used for response-level descriptive comparisons. T0 was partly retrospective and was not treated as a prospective baseline.
A. Contextual fields	Primary and nurse-specific questionnaires	All available respondents, depending on dataset	Eta	Age group	Categorical descriptor	Age bands as exported in the source files.	Used only for descriptive/contextual checks; not linked to unique individuals.
A. Contextual fields	Nurse-specific questionnaire	International nurses	Esperienza	Years of professional experience	Categorical descriptor	Experience bands as exported in the source files.	Used only for descriptive/contextual checks; not linked to unique individuals.
B. Primary monitoring items	Primary multi-stakeholder monitoring questionnaire	Clinical coaches; ward team; international nurses	LINGUA	Language proficiency	Single five-point monitoring item	Five-point ordered response scale; higher scores indicate more favourable perceived adequacy. Original verbal anchor labels were not present in the analysis-ready export.	Analyzed as an ordinal response-level item. Threshold >=3 was used pragmatically for perceived adequacy; threshold >=4 indicated a clearly favourable rating.
B. Primary monitoring items	Primary multi-stakeholder monitoring questionnaire	Clinical coaches; ward team; international nurses	FORMAZIONE	Professional training/competence	Single five-point monitoring item	Five-point ordered response scale; higher scores indicate more favourable perceived adequacy. Original verbal anchor labels were not present in the analysis-ready export.	Analyzed as an ordinal response-level item. Threshold >=3 was used pragmatically for perceived adequacy; threshold >=4 indicated a clearly favourable rating.
C. Nurse-specific welcome and retention items	Nurse-specific questionnaire	International nurses	ASST_accoglienza	Welcome from the organization	Single five-point welcome item	Five-point ordered response scale; higher scores indicate a more favourable perceived welcome.	Used descriptively and as one component of the overall welcome composite.
C. Nurse-specific welcome and retention items	Nurse-specific questionnaire	International nurses	Coord_accoglienza	Welcome from the nurse coordinator	Single five-point welcome item	Five-point ordered response scale; higher scores indicate a more favourable perceived welcome.	Used descriptively and as one component of the overall welcome composite.
C. Nurse-specific welcome and retention items	Nurse-specific questionnaire	International nurses	Equipe_inf_OSS_accoglienza	Welcome from the ward team	Single five-point welcome item	Five-point ordered response scale; higher scores indicate a more favourable perceived welcome.	Used descriptively and as one component of the overall welcome composite.
C. Nurse-specific welcome and	Nurse-specific questionnaire	International nurses	Medici_accoglienza	Welcome from physicians	Single five-point welcome item	Five-point ordered response scale; higher scores indicate a more favourable perceived welcome.	Used descriptively and as one component of the overall welcome composite.

Panel	Dataset / questionnaire	Respondent group	Source field(s)	English analytic label	Item / composite structure	Response anchors / coding	Scoring and analysis use
retention items							
C. Nurse-specific welcome and retention items	Nurse-specific questionnaire	International nurses	ACC_MEAN	Overall welcome	Composite score calculated as the arithmetic mean of the four welcome items from organization, coordinator, ward team, and physicians when available; no imputation was performed. In the main nurse-specific analytic subset, all four component items were available for all 14 response rows.	Composite retains the 1-5 metric when all component items are on the same five-point ordered response scale; decimal values are possible.	Used as the primary welcome summary for descriptive statistics and Spearman correlations with retention-related outcomes.
C. Nurse-specific welcome and retention items	Nurse-specific questionnaire	International nurses	Retention / Retention0	Intention to stay	Single five-point retention-related item	Five-point ordered response scale; higher scores indicate stronger intention to stay.	Used descriptively and in Spearman correlations with welcome-related variables.
C. Nurse-specific welcome and retention items	Nurse-specific questionnaire	International nurses	Satisfaction	Satisfaction	Single five-point satisfaction item	Five-point ordered response scale; higher scores indicate greater satisfaction.	Used descriptively and in Spearman correlations with welcome-related variables.
C. Nurse-specific welcome and retention items	Nurse-specific questionnaire	International nurses	Diffusione_ESTERO	Willingness to recommend the experience	Single five-point advocacy/recommendation item	Five-point ordered response scale; higher scores indicate stronger willingness to recommend the experience.	Used descriptively and in Spearman correlations with welcome-related variables.
C. Nurse-specific welcome and retention items	Nurse-specific questionnaire	International nurses	Autonomia / Percezione Autonomia	Perceived autonomy	Single five-point autonomy item when numerically available	Five-point ordered response scale when numeric; some source rows were coded as ND.	Documented as a questionnaire field. It was not used as a main reported outcome when numeric data were unavailable or inconsistent in the analytic subset.

Interpretation notes: primary LINGUA and FORMAZIONE items were self-ratings for international nurses and proxy ratings for clinical coaches and ward teams. Pooled analyses were therefore interpreted as organizational response-level summaries rather than estimates of individual nurse change. Overall welcome was treated as a simple arithmetic mean, not as a psychometrically validated scale score.
