# Supplementary material for: Structured Onboarding of International Nurses in an Italian Healthcare Organization: An Exploratory Multi-Stakeholder Pilot and Feasibility Observational Study
Source: Healthcare (Basel). 2026 Aug 15;14(16):2557. doi: 10.3390/healthcare14162557 (PMC13512028; doi:10.3390/healthcare14162557)
Supplement: Supplementary file 1 [file healthcare-14-02557-s001.zip › Supplementary_Table_S2_stakeholder_primary_descriptives.pdf]

Supplementary Table S2. Stakeholder-specific primary descriptive summaries by domain and conceptual timepoint

Values are response-level summaries from the anonymous pilot dataset. Percentages indicate ratings at or above the pragmatic adequacy threshold (>=3) and clearly favorable threshold (>=4) on the five-point response scale. Stakeholder-specific rows should not be interpreted as paired individual trajectories.

Domain	Stakeholder group	Timepoint	n	Median (IQR)	Mean (SD)	>=3	>=4
Language proficiency	Clinical coaches	T0	11	2 (1-3)	1.91 (0.94)	36.4%	0.0%
Language proficiency	Clinical coaches	T1	11	3 (2-3)	2.73 (1.19)	63.6%	18.2%
Language proficiency	Clinical coaches	T2	15	3 (3-4)	3.47 (0.74)	93.3%	46.7%
Language proficiency	International nurses	T0	14	4 (3-4)	3.79 (0.7)	100.0%	64.3%
Language proficiency	International nurses	T1	14	3 (3-3)	3.07 (0.27)	100.0%	7.1%
Language proficiency	International nurses	T2	12	4 (3-4)	3.75 (0.75)	100.0%	58.3%
Language proficiency	Ward team	T0	102	2 (2-3)	2.4 (1.01)	49.0%	11.8%
Language proficiency	Ward team	T1	102	3 (2.25-4)	3.04 (1.05)	74.5%	34.3%
Language proficiency	Ward team	T2	129	3 (3-4)	3.28 (0.73)	89.9%	36.4%
Professional training/competence	Clinical coaches	T0	11	3 (2-3)	2.46 (0.69)	54.5%	0.0%
Professional training/competence	Clinical coaches	T1	11	3 (2-3.5)	3 (1)	63.6%	27.3%
Professional training/competence	Clinical coaches	T2	15	3 (3-4)	3.2 (0.78)	80.0%	40.0%
Professional training/competence	International nurses	T0	14	4 (4-4)	4 (0.68)	100.0%	78.6%
Professional training/competence	International nurses	T1	14	4 (3-4)	3.71 (0.73)	100.0%	57.1%
Professional training/competence	International nurses	T2	12	4 (3.75-4.25)	4 (0.74)	100.0%	75.0%
Professional training/competence	Ward team	T0	102	2 (2-3)	2.58 (1.07)	47.1%	24.5%
Professional training/competence	Ward team	T1	102	3 (3-4)	3.11 (1.05)	76.5%	34.3%
Professional	Ward team	T2	129	3 (3-4)	3.36 (0.79)	89.9%	39.5%

Domain	Stakeholder group	Timepoint	n	Median (IQR)	Mean (SD)	>=3	>=4
training/competence							

Abbreviations: IQR, interquartile range; SD, standard deviation. T0 was partly retrospective and should not be interpreted as a prospective baseline.
