# Supplementary material for: Structured Onboarding of International Nurses in an Italian Healthcare Organization: An Exploratory Multi-Stakeholder Pilot and Feasibility Observational Study
Source: Healthcare (Basel). 2026 Aug 15;14(16):2557. doi: 10.3390/healthcare14162557 (PMC13512028; doi:10.3390/healthcare14162557)
Supplement: Supplementary file 1 [file healthcare-14-02557-s001.zip › Supplementary_Table_S3_paired_T0_T1_sensitivity.pdf]

Supplementary Table S3. Paired T0-T1 sensitivity summary for primary response-level outcomes

This table summarizes T0-T1 paired sensitivity results for the two primary monitoring domains. The analysis should be interpreted only as an exploratory sensitivity check. Stable observed identifiers were not available to verify unique nurses, link T2 to the first wave, or support confirmatory longitudinal inference.

Panel A. Overall pooled response-level sensitivity summary

Stakeholder group	Domain	Paired n	T0 mean	T1 mean	Mean delta	Median delta (IQR)	HL shift (95% CI)	Improved / unchanged / worsened	Wilcoxon signed-rank p	Sign-test p
All stakeholder responses	Language proficiency	127	2.51	3.02	0.50	0.0 (-1.0 to 1.0)	0.5 (0.0 to 0.5)	59 (46.5%) / 35 (27.6%) / 33 (26.0%)	<0.001	0.009
All stakeholder responses	Professional training/competence	127	2.72	3.17	0.44	0.0 (-1.0 to 2.0)	0.5 (0.0 to 0.5)	61 (48.0%) / 27 (21.3%) / 39 (30.7%)	0.003	0.035

Panel B. Stakeholder-specific sensitivity summary

Stakeholder group	Domain	Paired n	T0 mean	T1 mean	Mean delta	Median delta (IQR)	HL shift (95% CI)	Improved / unchanged / worsened	Wilcoxon signed-rank p	Sign-test p
Clinical coaches	Language proficiency	11	1.91	2.73	0.82	0.0 (0.0 to 1.5)	0.5 (-0.0 to 1.5)	5 (45.5%) / 5 (45.5%) / 1 (9.1%)	0.089	0.219
International nurses	Language proficiency	14	3.79	3.07	-0.71	-1.0 (-1.0 to 0.0)	-1.0 (-1.0 to 0.0)	1 (7.1%) / 4 (28.6%) / 9 (64.3%)	0.015	0.021
Ward team	Language proficiency	102	2.40	3.04	0.64	1.0 (0.0 to 2.0)	0.5 (0.5 to 1.0)	53 (52.0%) / 26 (25.5%) / 23 (22.5%)	<0.001	<0.001
Clinical coaches	Professional training/competence	11	2.45	3.00	0.55	0.0 (0.0 to 1.5)	0.5 (-0.5 to 1.5)	5 (45.5%) / 4 (36.4%) / 2 (18.2%)	0.141	0.453
International nurses	Professional training/competence	14	4.00	3.71	-0.29	0.0 (-1.0 to 0.8)	0.0 (-1.0 to 0.5)	4 (28.6%) / 5 (35.7%) / 5 (35.7%)	0.326	1.000
Ward team	Professional training/competence	102	2.58	3.11	0.53	1.0 (-1.0 to 2.0)	0.5 (0.0 to 1.0)	52 (51.0%) / 18 (17.6%) / 32 (31.4%)	0.002	0.037

Abbreviations: HL, Hodges-Lehmann. T0 refers to the arrival or early-days rating collected retrospectively in the first wave; T1 refers to the approximately 3-month rating. Delta = T1 - T0; positive values indicate more favourable T1 ratings. P values are descriptive screening information only and should not be interpreted as evidence of causal or within-person change.
